# Acute kidney injury and acyclovir-associated encephalopathy after administration of valacyclovir in an elderly person with normal renal function: A case report and literature review

**DOI:** 10.1097/MD.0000000000026147

**Published:** 2021-05-28

**Authors:** Tsuneaki Kenzaka, Kazuma Sugimoto, Ken Goda, Hozuka Akita

**Affiliations:** a Department of Internal Medicine, Hyogo Prefectural Tamba Medical Center, Tamba; b Division of Community Medicine and Career Development, Kobe University Graduate School of Medicine, Kobe, Japan.

**Keywords:** acute kidney injury, acyclovir neurotoxicity, case report, herpes zoster, valacyclovir

## Abstract

**Introduction:**

Acyclovir (ACV)-associated encephalopathy is related to an increase in plasma levels of 9-carboxymethoxymethylguanine, an ACV metabolite, and is often reported in patients with renal dysfunction. We report a case of ACV-associated encephalopathy with rapid progression of renal dysfunction after oral administration of valacyclovir (VACV) and review literature of previous ACV-associated encephalopathy cases.

**Patient concerns:**

An 88-year-old man was diagnosed with herpes zoster. VACV (3000 mg/day) treatment was initiated. Serum creatinine (Cr) level was 0.80 mg/dL. However, irritability, memory impairment, and decreased responsiveness occurred after 3 days. The Cr level was 6.76 mg/dL on admission.

**Diagnosis:**

He was diagnosed with ACV-associated encephalopathy with acute kidney injury.

**Interventions:**

VACV was discontinued, hemodialysis was initiated on the day of admission, and then the signs and symptoms improved approximately 72 hours after the admission.

**Conclusion:**

Worsening of renal function and encephalopathy should be a focus when using VACV or ACV, regardless of age and original renal function. Acute kidney injury and ACV-associated encephalopathy may particularly occur in the elderly even when renal function is normal. Therefore, regular monitoring of renal function and consciousness is necessary during VACV treatment.

## 1 Introduction

Acyclovir (ACV) associated encephalopathy is a very rare case induced by ACV and valacyclovir (VACV), a prodrug of ACV.^[1]^ Rashiq et al reported that several neuropsychiatric symptoms, such as consciousness disturbance, tremor, and myoclonus, usually occur within 2 days of administering VACV.^[1]^ Hallucination frequently occurs in addition to consciousness disturbance and involuntary movements.^[1–3]^ However, headache, fever, convulsions, and focal symptoms are rare.^[2]^ Abnormalities in cerebrospinal fluid examinations or head computed tomography (CT)/magnetic resonance imaging are generally not observed, and symptoms disappear 48 to 72 hours after discontinuing ACV. However, dialysis may be necessary.^[4]^

ACV-associated encephalopathy is related to an increase in plasma levels of 9-carboxymethoxymethylguanine, a metabolite of ACV, and is often reported in patients with renal dysfunction.^[5]^ However, there are few reports of the onset of ACV-associated encephalopathy in patients in whom renal dysfunction was not indicated.^[4]^ Here, we report a case of ACV-associated encephalopathy with the rapid progression of renal dysfunction after oral VACV administration, although serum creatinine (Cr) levels were normal. In addition, we report a review of previous ACV-associated encephalopathy cases.

## 2 Case report

An 88-year-old man who could independently perform activities of daily living visited the hospital with a primary complaint of consciousness disturbance. The patient had a history of radical resection for prostate cancer (T1c N0 M0, stage I). His history of varicella-zoster infection was unknown. A painful vesicular eruption appeared in the right axilla 8 days before admission, and he visited a nearby clinic the following day. The Cr level was 0.80 mg/dL. He was consequently diagnosed with herpes zoster. Thus, VACV administration (3000 mg/d) was initiated. Pregabalin (75 mg/d) and mecobalamin (1500 μg/d) were also administered for analgesic purposes without the concomitant use of nonsteroidal anti-inflammatory drugs. The patient experienced pain that led to reduced food intake and dehydration. He urinated about 4 times daily. Also, irritability, memory impairment, and decreased responsiveness occurred after 3 days, and the patient was admitted to our hospital for emergency treatment due to exacerbated symptoms.

Physical findings on admission were as follows: E3V3M6 on the Glasgow Coma Scale, body temperature of 35°C, blood pressure of 110/60 mm Hg, pulse rate of 60 beats/min, respiratory rate of 20 breaths/min, and oxygen saturation level of 96% (room air). The patient had xerostomia. Herpes zoster scarring on the right upper limb (TH-1/TH-2 areas) was noted in the extremities. Furthermore, examination of meningeal irritation symptoms showed no neck stiffness, negative Kernig sign, and negative Brudzinski sign. The diameter/light reflex of the pupils was 2+/2+. Myoclonus was observed with no clear paralysis. Hematologic examination results were as follows: white blood cell, 6530/μL; C-reactive protein, 1.07 mg/dL; blood urea nitrogen, 58.4 mg/dL; Cr, 6.76 mg/dL; and blood glucose, 91 mg/dL (Table 1). The urine sediment showed muddy brown casts of epithelial cells, indicating acute tubular necrosis. Cerebrospinal fluid test results revealed an initial pressure of 13 cm H_2_O, cell count of 71/μL (mononuclear cell count, 70/μL), protein level of 147 mg/dL, and glucose level of 48 mg/dL (Table 2). However, blood, urine, and cerebrospinal fluid cultures were negative.

**Table 1:** Laboratory data on admission.

Parameter	Recorded value	Standard value
White blood cell count	6530/μL	4500–7500/μL
Neutrophils	68%	42%–74%
Hemoglobin	11.7 g/dL	11.3–15.2 g/dL
Hematocrit	34.2%	36%–45%
Platelet count	17.0 × 10^4^/μL	13–35 × 10^4^/μL
International normalized ratio	0.93	0.80–1.20
Activated partial thromboplastin time	23.3 s	26.9–38.1 s
Fibrin degradation products	10.4 μg/mL	2.0–8.0 μg/mL
C-reactive protein	1.07 mg/dL	<0.14 mg/dL
Estimated glomerular filtration rate	6.6	
Total protein	6.6 g/dL	6.9–8.4 g/dL
Albumin	3.5 g/dL	3.9–5.1 g/dL
Total bilirubin	0.3 mg/dL	0.2–1.2 mg/dL
Aspartate aminotransferase	25 U/L	11–30 U/L
Alanine aminotransferase	8 U/L	4–30 U/L
Lactate dehydrogenase	227 U/L	109–216 U/L
Creatine phosphokinase	252 U/L	40–150 U/L
Blood urea nitrogen	58.4 mg/dL	8–20 mg/dL
Creatinine	6.76 mg/dL	0.63–1.03 mg/dL
Sodium	130 mEq/L	136–148 mEq/L
Potassium	6.4 mEq/L	3.6–5.0 mEq/L
Glucose	91 mg/dL	70–109 mg/dL
Hemoglobin A1c	5.4%	<6.5%
Thyroid-stimulating hormone	3.022 IU/mL	0.541–4.261 μIU/mL
Free thyroxine	0.9 ng/dL	0.72–1.51 ng/dL
Ammonia	35 g/dL	12–66 g/dL
ACV	34.6 g/mL	
pH	7.359	7.350–7.450
Partial pressure of arterial carbon dioxide	37.3 mm Hg	35–45 mm Hg
Partial pressure of arterial oxygen	88.6 mm Hg	80–100 mm Hg
Bicarbonate	21.6 mEq/L	22–26 mEq/L
Lactate	1.26 mmol/L	<2.0 mmol/L

**Table 2:** Results of cerebrospinal fluid tests on admission.

Parameter	Recorded value	Standard value
Cell count	71/μL	0–5/μL
Mononuclear count	70/μL	
Polynuclear count	1/μL	
Total protein	147 mg/dL	10–40 mg/dL
Glucose	48 mg/dL	50–75 mg/dL
Lactate dehydrogenase	39 IU/L	0–25 IU/L
Creatine phosphokinase	3 IU/L	<6 IU/L
HSV DNA PCR	Negative	
VZV DNA PCR	Negative	

The hemodynamics were maintained, but ultrasound showed that the inferior vena cava collapsed, suggesting dehydration. Abdominal CT revealed no obstruction, and postrenal renal failure was ruled out. The maximum diameter of the kidney was 62 mm on the right and 65 mm on the left, and there was no prominent renal swelling. However, urinary retention of about 250 mL in the bladder was observed. Moreover, head magnetic resonance imaging did not reveal any findings suggestive of encephalitis.

**Figure 1:**
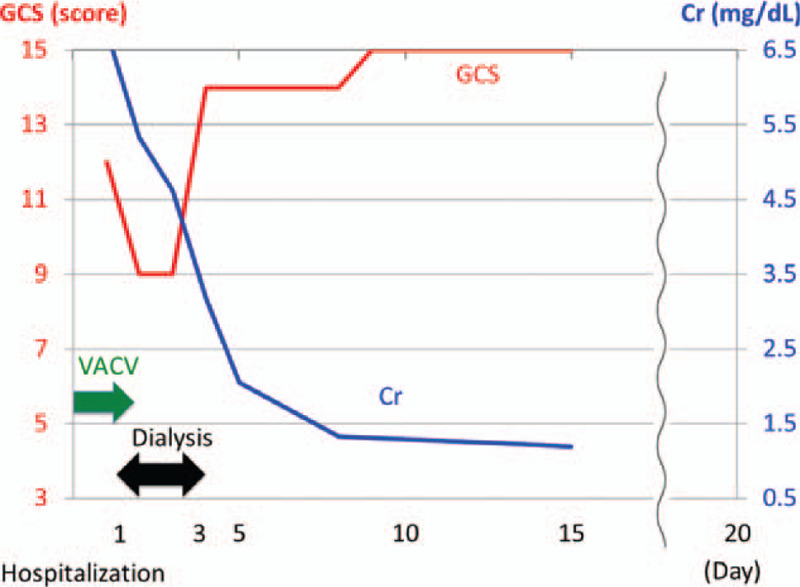
Clinical course.

Table 3 shows the comparison of ACV-associated encephalopathy and varicella zoster virus encephalitis.^[6,7]^ Our elderly patient had taken VACV for a sufficient period and was thus suspected to have ACV-associated encephalopathy based on the absence of fever, stiff neck, and headache, and normal imaging findings. The clinical course is shown in Fig. 1. VACV was discontinued, hemodialysis was initiated from the day of admission to day 3, and then the signs and symptoms improved approximately 72 hours after the admission. The Glasgow Coma Scale score was 14 points, and hemodialysis was discontinued on hospital day 4. The plasma concentration of ACV level at the time of examination, which was discovered later, was markedly elevated (34.6 μg/mL), and results of polymerase chain reaction analysis of the cerebrospinal fluid were negative for herpes simplex virus and varicella zoster virus DNA. The plasma concentration of ACV level was <0.5 μg/mL (normal range <2.0) when the consciousness level became normal on day 10 of hospitalization. Negative blood, urine, and cerebrospinal fluid cultures ruled out bacterial consciousness disorder. Furthermore, the consciousness level did not improve immediately after the dialysis on day 1; however, it improved after the dialysis was performed for 3 days. Therefore, the consciousness disorder due to uremia was ruled out. Thus, a definitive diagnosis of ACV-associated encephalopathy was made based on the patient's course. The increase in cell count in the cerebrospinal fluid could have been due to the effects of ACV-associated encephalopathy, although this finding was atypical.

**Table 3:** Differences between acyclovir-associated encephalopathy and varicella zoster virus encephalitis.

	ACV-associated encephalopathy	VZV encephalitis
Risk factors	ACVElderlyNSAIDs	ImmunocompromisedCranial nerve dermatomePresence of cutaneous dissemination
Symptoms	Rarely meningismus·fever·headache	Meningismus·fever·headache
Cerebrospinal fluid	Normal	Lymphocyte domination
Imaging studies	Normal	Abnormal (50%)
Treatment	ACV discontinuedDialysis	ACV
Prognosis	Improve (within 48–72 h)	Mortality 0%–25%(Normal immunity)

Subsequently, ambulatory discharge was possible on hospital day 35 without any sequelae.

### 2.1 Search strategy

The terms “acyclovir neurotoxicity” or “acyclovir encephalopathy” were searched in the MEDLINE database. Fifty-one cases have existed in the literature since 1988. Among those 51 cases, 35 reported acyclovir neurotoxicity when limited to the English and Japanese literature.

## 3 Discussion

We report a case of ACV-associated encephalopathy with rapid progression of renal dysfunction after oral VACV administration despite normal serum Cr levels (0.80 mg/dL). The patient experienced pain that led to reduced food intake and dehydration. Moreover, the use of VACV, which has a high oral bioavailability and a long plasma half-life, caused renal dysfunction, leading to ACV-associated encephalopathy. Furthermore, as shown in Table 4, ACV-associated encephalopathy may occur even under normal renal function or prophylactic administration of antiviral drugs. ACV-associated encephalopathy is commonly observed in patients with impaired renal function but may develop even when renal function is normal.^[1]^

**Table 4:** A summary of the literature review on ACV-associated encephalopathy cases.

Case	Author	Reference number	Age	Sex	Cause	Medication (dosing period, days)	Total dosing period (days)	Dosage (mg/day)	Comorbidity	Serum acyclovir measurement	Dialysis treatment for underlying disease	Normal Creatinine (mg/dL)	Onset Creatinine (mg/dL)	Concomitant drug
1	Umoru GO et al	^[9]^	57	Man	Herpes Zoster	Oral ACV (4)	4	4000 mg/d	hemodialysis, type2 diabetes mellitus, hypertension, hyperlipidemia, coronary artery disease, congestive heart failure, shingles, and multiple incision and drainage procedures for bilateral recurrent abscesses in his thighs	Yes	HD	Unknown	Unknown	Unknown
2	Kawabe Matsukawa M et al	^[10]^	77	Man	Herpes Zoster	Oral ACV (2)	2	800 mg/d	Angina after stenting, Hyperuricemia, Dyslipidemia, hypertension	No	CAPD	9∼10	12.58	Unknown
3	Bełz A et al	^[11]^	86	Unknown	Herpetic simplex keratitis	Oral VACV (4)	4	800 mg/d	chronic heart failure Class IINYHA (ejection fraction 40%), moderate mixed aortic valve disease, mild mitral and tricuspid insufficiency, paroxysmal atrial fibrillation, and type 2 diabetes mellitus	No	HD	Unknown	8.48	Unknown
4	Ikuta K et al	^[12]^	27	Man	Herpes simplex virus–1	intravenous ACV (6)→intravenous ACV (12)	18	30 mg/kg/d (6 days)→15 mg/kg/d	Hepatitis A infection, Hepatitis B infection	Yes	None	1.4	None	None
5	Patel J et al	^[13]^	63	Man	Herpes Zoster	Oral ACV (5)	5	4000 mg/d	Abscesses in his thighs	No	None	1	1.2	Unknown
6	Sadjadi SA et al	^[14]^	80	Man	Herpes Zoster	intravenous ACV(2)→oral ACV(1)	3	5 mg/kg/d→200 mg/ds	Hypertension, congestive heart failure and end stage renal disease	Yes	CAPD	None	None	Unknown
7	Gorlitsky BR et al	^[15]^	60	Man	Herpes simplex virus–2	Oral VACV (4)	4	800 mg/d	hypertensive nephrosclerosis and diabetes	Yes	HD	None	9.73	Unknown
8	Watson WA et al	^[16]^	62	Man	Herpes Zoster	Oral VACV (14)→intravenous ACV (2)→intravenous ACV (6)	16	Unknown→Unknown→24.2 mg/kg	Goodpasture syndrome complicated by end-stage renal disease requiring a living donor kidney transplant 11 years prior to presentation, chronic allograft glomerulopathy, and a recent diagnosis of collagenous colitis.	No	None	1.2	2.5	Unknown
9	Thind GS et al	^[17]^	82	Man	Herpes Zoster	Oral VACV (5)→intravenous ACV (6)	11	3000 mg/d→5 mg/kg	type 2 diabetes mellitus, a history of coronary artery disease, chronic atrial fibrillation, gastro-esophageal reflux disease and gout	No	HD	None	None	None
10	Chowdhury MA et al	^[5]^	69	Woman	Herpes simplex virus	Intravenous ACV (1.5)	1.5	1500 mg/d	hypertension, diabetes, chronic obstructive pulmonary disease, and end-stage renal disease on hemodialysis was admitted with a diagnosis of pneumonia and right breast cellulitis	Yes	HD	None	None	None
11	Sacchetti D et al	^[4]^	69	Woman	Herpes zoster	Oral ACV (2)→intravenous ACV (1)→intravenous ACV (2)	5	800 mg/d→1500 mg/d→550 mg/d	uncontrolled diabetes and asthma	No	None	Unknown	3.94	NSAIDs
12	Adair JC et al	^[3]^	70	Woman	Herpes zoster	Oral ACV (2)	2	1400 mg/d	Granulomatosis with polyangiitis	Yes	HD	Unknown	Unknown	Unknown
13	Adair JC et al	^[3]^	64	Woman	Herpes simplex virus	Oral ACV (2)	2	600 mg/d	hemolytic uremic syndrome	No	HD	8.8	None	Unknown
14	Tomori K et al	^[18]^	30	Woman	Herpes simplex virus	intravenous ACV (2)	2	1000 mg/d	None	No	None	Unknown	Unknown	Unknown
15	Itoh M et al	^[19]^	7	Woman	Herpes simplex virus	Oral ACV (2)	2	1000 mg/d	None	No	None	Unknown	0.3	Unknown
16	Gómez Campderá FJ et al	^[20]^	59	Woman	Herpes zoster	Oral ACV (7)	7	200 mg/d	secondary to chronic interstitial nephropathy.	Yes	HD	Unknown	Unknown	Unknown
17	Hoskote SS et al	^[21]^	52	Man	Herpes zoster	Oral VACV (7)→oral ACV (2)→intravenous ACV (6)	15	3000 mg/d→1000 mg/d→600 mg/d	hypertension, diastolic congestive heart failure, end-stage renal disease on hemodialysis 3 times a week, hemorrhagic stroke	No	HD	Unknown	Unknown	Unknown
18	Sagawa N et al	^[22]^	83	Man	Herpes zoster	Oral VACV (5)	5	3000 mg/d	type 2 diabetes mellitus	Yes	None	0.8	5.11	NSAIDs
19	Strong DK et al	^[23]^	5	Woman	Epstein-Barr virus-induced lymphoproliferative disease	Intravenous ACV (2)→intravenous ACV (12)	14	920 mg/m^2^/d→460 mg/m2 3 times wk	cadaveric renal transplant for end-stage renal failure due to cystinosis	Yes	None	Unknown	Unknown	Unknown
20	Blohm ME et al	^[24]^	12	Woman	Prevention	Intravenous ACV (8)→intravenous ACV (18)	26	30 mg/kg→20 mg/kg	CML	Yes	None	0.8	1.7	Unknown
21	Peces R *et al*	^[25]^	44	Man	Herpes zoster	Oral ACV (2)	2	4800 mg/d	Unknown	No	HD	Unknown	Unknown	Unknown
22	Mesar I et al	^[26]^	78	Woman	Herpes zoster	Oral ACV (2)	2	4000 mg/d	Endemic nephropathy, arterial hypertension, cardiovascular disease	No	HD	Unknown	Unknown	Unknown
23	Mesar I et al	^[26]^	61	Man	Herpes zoster	ACV (Unknown)	Unknown	Unknown	Extracapillary glomerulonephritis, arterial hypertension	No	HD	Unknown	Unknown	Unknown
24	Mesar I et al	^[26]^	72	Woman	Herpes zoster	Oral VACV (4)	3	1600 mg/d	renal amyloidosis, arterial hypertension, hypothyroidism	No	HD	Unknown	Unknown	Unknown
25	Asahi T et al	^[2]^	78	Woman	Herpes zoster	Oral VACV (5)	5	3000 mg/d	Alzheimer's disease	No	None	Unknown	3.2	Unknown
26	Asahi T et al	^[2]^	73	Man	Herpes zoster	Oral ACV (2)	2	3000 mg/d	chronic renal failure	No	HD	Unknown	Unknown	Unknown
27	Hussein MM et al	^[27]^	51	Man	Anti-CMV prophylaxis	Oral GCV (5)	5	1.25 mg/d every 48 h	end-stage renal disease of uncertain etiology, diabetes mellitus	No	HD	Unknown	10.45	Unknown
28	Yang HH et al	^[28]^	70	Man	Herpes zoster	Intravenous ACV (1.5)	1.5	500 mg/d	rectal cancer status post-colostomy and end-stage renal disease	Yes	HD	5.7	6.2	Unknown
29	Chevret L et al	^[29]^	0.5	Woman	Prevention	Intravenous ACV (2)→intravenous ACV (1)	3	250 mg/m^2^→750 mg/m^2^	Acute liver failure, related to neonatal enterovirus infection, occurred within a few days after birth, liver transplantation at 6 months of age	Yes	None	Unknown	Unknown	Unknown
30	Peyrière H et al	^[30]^	13	Man	Prevention	Intravenous GCV (14)+VGCV(Unknown)→oral ACV(2)→oral VGCV(Unknown)	16	Unknown→450 mg/d (every 2 d)→600 mg/d→450 mg/d (twice weekly)	acute lymphoblastic leukemia	Yes	None	Unknown	Unknown	Unknown
31	Rajan GR et al	^[31]^	73	Man	Herpes simplex labialis	Intravenous ACV (2)	2	400 mg/d	amiodarone pulmonary toxicity, coronary artery bypass grafting, chronic atrial fibrillation, non-sustained ventricular tachycardia, and congestive heart failure	Yes	None	Unknown	Unknown	Unknown
32	Beales P et al	^[32]^	51	Man	Herpes zoster	Oral ACV (1.5)	1.5	1600 mg/d	end-stage renal failure due to IgA nephropathy, poor blood pressure control	Yes	HD	Unknown	Unknown	Unknown
33	Beales P et al	^[32]^	56	Woman	Herpes zoster	Oral ACV (9)	9	1600 mg	end-stage renal failure of uncertain cause, tuberculosis, lumbar osteomyelitis, and recurrent continuous ambulatory peritoneal dialysis peritonitis	Yes	HD	Unknown	Unknown	Unknown
34	Krieble BF et al	^[33]^	77	Woman	Herpes zoster	Intravenous ACV (2)	2	3000 mg/d	None	Yes	None	1.09	4.46	None
35	Davenport A et al	^[34]^	72	Woman	Herpes zoster	Oral ACV (1)→intravenous ACV (1)→intravenous+oral ACV (1)	1	800 mg/d→4 mg/kg/d→4 mg/kg/d+800 mg/d	end-stage renal failure due to chronic pyelonephritis	Yes	CAPD	Unknown	Unknown	Unknown
36	Davenport A et al	^[34]^	41	Man	Viral pneumonia	Oral ACV (5)	5	1600 mg/d	end-stage renal failure secondary to focal glomerular sclerosis	Yes	CAPD	Unknown	Unknown	Unknown
37	MacDiarmaid-Gordon AR et al	^[35]^	62	Man	Herpes zoster	Oral ACV(Unknown)	Unknown	2000 mg/d	None	No	CAPD	Unknown	Unknown	Unknown
38	MacDiarmaid-Gordon AR et al	^[35]^	47	Man	Herpes zoster	Oral ACV (3)	3	4000 mg/d	Unknown	No	HD	Unknown	Unknown	Unknown
39	MacDiarmaid-Gordon AR et al	^[35]^	30	Man	Herpes zoster	Oral ACV (3)→oral ACV (5)	8	2000 mg/d→1000 mg/d	Granulomatosis with polyangiitis	No	HD	Unknown	Unknown	Unknown
40	MacDiarmaid-Gordon AR et al	^[35]^	56	Man	Herpes zoster	Oral ACV (9.2)	9	2000 mg/d	Unknown	Yes	HD	Unknown	Unknown	Unknown
41	Swan SK et al	^[36]^	76	Woman	Herpes zoster	Oral ACV (4)	4	1000 mg/d	Unknown	No	HD	Unknown	Unknown	Unknown
42	Feldman S et al	^[37]^	17	Woman	Herpes simplex virus	Intravenous ACV (2)	2	4000 mg/d	metastatic ovarian germ cell tumor	Yes	None	1.5	Unknown	Unknown
43	Sugimoto K et al	^[38]^	70	Man	Prevention	Oral VACV (36)	36	500 mg three times a wk	multiple myeloma	No	None	8.78	7.71	None

Two mechanisms of ACV-induced acute kidney injury exist. One is renal dysfunction due to dehydration and the use of nonsteroidal anti-inflammatory drugs, as well as tubular obstruction due to ACV itself,^[5]^ and the other is renal dysfunction caused by a direct mechanism of ACV aldehyde.^[6]^ The serum ACV level increases due to dysuria when renal dysfunction occurs, which further exacerbates renal dysfunction and causes ACV-associated encephalopathy.^[8]^ Elderly people are prone to dehydration and potentially impaired renal function. The aforementioned mechanism causes acute renal damage and a tendency for the onset of ACV-associated encephalopathy. Moreover, VACV is a prodrug of ACV and has better gastrointestinal absorption than ACV. Consequently, the oral bioavailability of ACV is 10% to 20% (54.2% for VACV), and its serum half-life is approximately 5 times longer. Hence, VACV is simpler to administer than ACV because the number of doses is smaller and characteristically tends to result in increased serum levels.^[1]^

In total, 43 cases of ACV-associated encephalopathy have been reported in 35 studies. A summary of the literature review is presented in Table 4. The age range of the patients with ACV-associated encephalopathy was from 0.5 to 88 years (mean age, 55.0 years; median age, 62 years). Among the patients, 24 (55.8%) were aged ≥60 years, and 6 (13.9%) were aged ≤18 years. The sex ratio was almost equal (18 females and 24 males [55.8]; 1 unknown). ACV-associated encephalopathy occurred following the treatment of herpes zoster in 27 cases (62.7%), treatment of herpetic simplex in nine cases (20.9%), and for the purpose of suppressing the onset of virus associated with chemotherapy in 5 cases (11.6%).

ACV-associated encephalopathy occurred in 24 patients (55.8%) using oral medication only. The administered antiviral agent was ACV in 37 cases (86.0%). The duration of antiviral administration was known in 40 patients, and the time of onset was 1 to 36 days (median, 4 days). Moreover, an NSAID was concomitantly used in only 2 patients (4.7%).

Many patients had an underlying disease, especially 27 dialysis patients (62.7%; 22 undergoing hemodialysis and 5 undergoing peritoneal dialysis). However, 4 patients (9.3%) had no underlying disease, and the presence of the underlying disease was unknown in 4 patients (9.3%).

Serum ACV concentration was measured in 21 of 43 cases (48.8%). The serum concentration of 9-carboxymethoxymethylguanine was measured in only 1 case (case 28). VACV is a prodrug of ACV, which becomes ACV in the blood; thus, there were no cases with VACV concentration measurement.

For many patients, the precritical serum Cr levels were unknown, and in 2 patients (4.7%), the levels were <1.0 mg/dL. Moreover, serum Cr levels at the time of onset were often unknown. The serum Cr level at the time of onset, when known, was elevated except in 1 patient, a 7-year-old child (0.3 mg/dL).

In conclusion, based on our case findings, it is important to focus on the worsening of renal function and encephalopathy when using VACV or ACV regardless of age and original renal function. Acute kidney injury and ACV-associated encephalopathy may particularly occur in the elderly even when renal function is normal. Therefore, regular monitoring of renal function and consciousness is necessary.

## Author contributions

**Conceptualization:** Tsuneaki Kenzaka.

**Data curation:** Tsuneaki Kenzaka, Ken Goda.

**Investigation:** Kazuma Sugimoto, Ken Goda.

**Supervision:** Hozuka Akita.

**Writing – original draft:** Tsuneaki Kenzaka.

**Writing – review & editing:** Kazuma Sugimoto, Ken Goda, Hozuka Akita.
